# Colorectal Cancer (CRC): Investigating the Expression of the Suppressor of Fused (*SuFu*) Gene and Its Relationship with Several Inflammatory Blood-Based Biomarkers

**DOI:** 10.3390/biomedicines11020540

**Published:** 2023-02-13

**Authors:** Tahseen Bilal Rather, Ishrat Parveiz, Gulzar A Bhat, Gowhar Rashid, Kulsum Akhtar, Rizwanul Haque, Mohammad Shamsul Ola, Mehboob Ali, Rauf A Wani, Ishrat Younas Khan, Syed Besina, Syed Mudassar

**Affiliations:** 1 Department of Clinical Biochemistry, Sher-I-Kashmir Institute of Medical Sciences, Soura, Srinagar 190011, India; 2 Scientist Multidisciplinary Research Unit, Sher-I-Kashmir Institute of Medical Sciences, Soura, Srinagar 190011, India; 3 Department of Amity Medical School, Amity University Haryana, Haryana 125001, India; 4 Department of Biotechnology, SEBES, Central University of South Bihar (Gaya), Bihar 824236, India; 5 Department of Biochemistry, College of Science, King Saud University, Riyadh 11451, Saudi Arabia; 6 Senior Scientist Toxicology Invivotek Nexus, a Genesis Biotech Group LLC Company, 17 Black Forest RD, Hamilton, NJ 08690, USA; 7 Department of General Surgery, Sher-I-Kashmir Institute of Medical Sciences, Soura, Srinagar 190011, India; 8 Department of Pathology, Sher-I-Kashmir Institute of Medical Sciences, Soura, Srinagar 190011, India

**Keywords:** *SuFu*, quantitative real-time PCR, immunohistochemistry, colorectal cancer, Kashmir

## Abstract

Background: Suppressor of fused (*SuFu*) is a tumor-suppressor gene that regulates hedgehog signaling. Its involvement in some malignancies is broadly accepted. However, its association with colorectal cancer (CRC) pathogenesis is not clear. Likewise, no study has clearly associated blood-based inflammatory biomarkers with cancer diagnosis/prognosis as yet. Aim: Our goal was to look at *SuFu* expression levels in CRC patients and its relationship with other clinicopathological factors. Additionally, we looked into the function of a few blood-based biomarkers in CRC and whether or not a combined strategy at the genetic and clinical levels can be applied in CRC. Methods: The investigation included 98 histopathologically confirmed CRC samples and adjacent normal tissues (controls). A colonoscopy was followed by a targeted biopsy for each suspected colon cancer patient. A CT scan and MRI were also performed on every patient with rectal cancer. Real-time polymerase chain reaction and immunohistochemistry (IHC) were used for assessment. A Beckman Coulter DxH900 was used to examine blood parameters. A Beckman Coulter DxI800 was used to identify pretreatment carcinoma embryonic antigens (CEA) and carbohydrate antigens (CA 19–9) in CRC patients. Results: The expression of *SuFu* was associated with gender, education, passive smoking, tumor grade, perineural invasion (PNI), lymph node metastasis (LNM), node status, stage, vital status, and recurrence (*p* < 0.05). In the combined analysis, the areas under the curve produced by the platelet-to-lymphocyte ratio (PLR), neutrophil-to-lymphocyte ratio (NLR), and red cell distribution width (RDW) were the greatest (AUC_RDW+PLR+NLR_ = 0.91, 95% CI: 0.86–0.93, *p* < 0.05). Furthermore, the most severe pathological features were linked to RDW, PLR, NLR, and HPR. *SuFu* expression, node status, LNM, PNI, and stage all had significant correlations with OS and DFS rates in IHC-based univariate survival analysis (*p* < 0.05). According to the Cox regression, CA-19.9 had a strong independent predictive link with 3-year DFS (*p* < 0.05). Conclusion: In CRC, *SuFu* was downregulated both transcriptionally and translationally, was primarily nucleo-cytoplasmic, and was expressed less in high-grade tumors. In addition, *SuFu* was linked to a poor overall and disease-free survival rate. It may be possible to use SuFu as a therapeutic target for CRC in the future. However, *SuFu* expression had no effect on RDW, PLR, NLR, or HPR serum levels.

## 1. Introduction

Worldwide, colorectal cancer (CRC) is the second leading cause of death [1]. Currently, surgery remains the primary treatment of choice at early stages but it does not benefit those with advanced cases. Hence, the disease has a poor prognosis. As of today, the standard treatment involves resection of the primary tumor as well as the lymph nodes, followed by adjuvant chemotherapy [2,3]. Like other malignancies, both genetic and modifiable environmental factors have been associated with CRC genesis [4,5,6,7]. Among the various genetic pathways, the hedgehog signaling pathway has been implicated in tumor formation in various organs [8]. However, until recently, investigations and research on the significance of the Hh signaling pathway in the development and progression of CRC have been divided. Its specific role in the genesis, progression, and spread of CRC is unknown. [9,10]. Suppressor of fused (*SuFu)* is one of the main downregulators of the hedgehog signaling pathway [11]. *SuFu*—a key tumor-suppressor gene [12] of the hedgehog signaling pathway—has been linked to various cancers [13,14,15]. An earlier study found that patients with gastric cancer had lower levels of *SuFu* expression [16]. Similarly, Li et al., in their previously conducted study, showed a tumor-suppressive role of *SuFu* in basal cell carcinoma [17]. However, its association with CRC is not conclusive. A cell line-based study showed that the overexpression of *SuFu* had a regulatory effect on colon cancer cells, and inhibited cell growth and tumor formation [18].

Additionally, several inflammatory biomarkers have recently been explored for their possible links with cancer as evidence suggests that there are links between inflammation and cancer progression [19,20]. Red cell distribution width (RDW) [21,22], hemoglobin-to-platelet ratio (HPR) [23], neutrophil-to-lymphocyte ratio (NLR) [24,25], and platelet-to-lymphocyte ratio (PLR) [26,27], for example, are among these markers. NRL, PLR, HPR, and RDW may assist in detecting the early stages of CRC. However, the data regarding their diagnostic value in CRC are not conclusive [28,29]. To date, no concrete work has been conducted regarding the expression status of *SuFu* in CRC, nor is much known about the utility of inflammatory blood biomarkers in CRC. To evaluate the role of *SuFu* in CRC development, we looked at its expression status and localization in tumor tissues as well as its possible links with important pathological features associated with metastasis, such as grade, stage, lymphovascular invasion (LVI), perineural invasion (PNI), lymph node metastasis, and distant metastasis (DM). Since there is currently no conclusive evidence linking blood-based inflammatory biomarkers with cancer diagnosis or prognosis, we further explored the potential of blood-based biomarkers as prognostic or diagnostic markers in CRC.

## 2. Methods

### 2.1. Patient Selection

This study included 98 human histopathologically confirmed CRC samples, as well as nearby normal tissues (controls). Colonoscopy and targeted biopsy were performed on all colon cancer patients. All patients with rectal cancer underwent abdominal CT and pelvic MRI. Between 2 April 2019 and 28 March 2022, samples were collected from patients who had undergone primary surgical resection at the Sher-I-Kashmir Institute of Medical Science (SKIMS) in the Department of General and Minimal Invasive Surgery. As recommended by the College of American Pathologists, the Royal College of Pathologists, the Commission on Cancer of the American College of Surgeons, and the National Cancer Institute [30], the tumor–node–metastasis (TNM) staging system of the American Joint Committee on Cancer (AJCC) [31] and the International Union Against Cancer (UICC) [32] was used for the staging of CRC. Although various criteria have been presented [33,34,35], the degree of gland formation is currently the most widely accepted and consistently used criterion for grading colorectal cancer (CRC). According to the TNM classification [36,37], a grade 1 tumor is one that has a high level of differentiation, grade 2 tumors have a moderate level of differentiation, grade 3 tumors have a low level of differentiation, and grade 4 tumors have no differentiation at all. For RNA extraction, samples were stored in RNAlater (Sigma-Aldrich Burlington, VT, USA) and kept at 80 °C for future processing. After receiving permission from the Department of Pathology, formalin-fixed paraffin-embedded (FFPE) blocks of CRC tissues and surrounding normal tissues were collected for immunohistochemistry examination (IHC). Tissues from the same CRC patients from whom fresh tissues had been taken at the time of surgery were used for the immunohistochemistry research. Further, the same patients whose tissues had already been employed in mRNA and protein expression underwent routine base-line investigations before surgery, and the data were collected electronically. Various blood parameters such as hemoglobin (HGB), red cell distribution width (RDW), white blood cells (WBC), platelets (PLT), neutrophil, and lymphocyte were recorded. An equal number of healthy controls were selected for blood analysis. A Beckman Coulter DxH900 was used to routinely analyze blood parameters: hemoglobin (HGB), red cell distribution width (RDW), white blood cells (WBC), platelets (PLT), neutrophil, and lymphocyte. NLR was calculated by dividing the absolute neutrophil count by the absolute lymphocyte count (ALC); likewise, PLR was computed by dividing the absolute platelet count by ALC. HPR was calculated as hemoglobin/total number of platelets. Pretreatment CEA and CA-19.9 levels in the CRC group were measured on using a Beckman Coulter DxI800. 

### 2.2. Data Collection

A structured questionnaire was administered to collect the data. We gathered information on family history, smoking status, socioeconomic status (SES), lifestyle, education, pesticide exposure, junk food, and intake of fruits and vegetables. Face-to-face interviews were conducted by a single author in order to reduce the interviewer bias. The term “family history” referred to whether or not the patient had a history of cancer in their own family or in a blood relative (both site-specific and other cancers). Regarding smoking status, we did not count how many packs of cigarettes patients smoked daily or weekly (frequency of smoking). We simply documented the patient’s statement of whether they smoked or did not smoke. To determine the patient’s socioeconomic position, the Kuppuswamy scale and BG Prasad scale [38], which are based on education, occupation, and total income, were employed. Despite being included in the questionnaire, we did not include different food intake measurements in the manuscript since there existed a strong unit measurement bias. Regarding lifestyle activity, sedentary behavior included occupations such as clerk, section officer, engineer, and other indoor occupations that were characterized by less energy expenditure, while active lifestyle occupations were characterized as having more effort and energy expenditure such as police officer, mechanic, farmer, construction worker, etc. However, we did not record any intensity level measurements. Concerning education status, we grouped illiterate, primary, and middle education into one group (lower) and higher secondary, graduate, and above into the higher group. Pesticide exposure was not quantified by us. If the patient had been exposed to pesticides, we merely recorded “yes” or “no” responses from them. Junk foods included those foods which were high in one or more components such as sugar, fat, cholesterol, salt, calories, etc., as described by chapman et al. [39], and usually prepared by deep frying.

### 2.3. Inclusion and Exclusion Criteria

**Figure 1:**
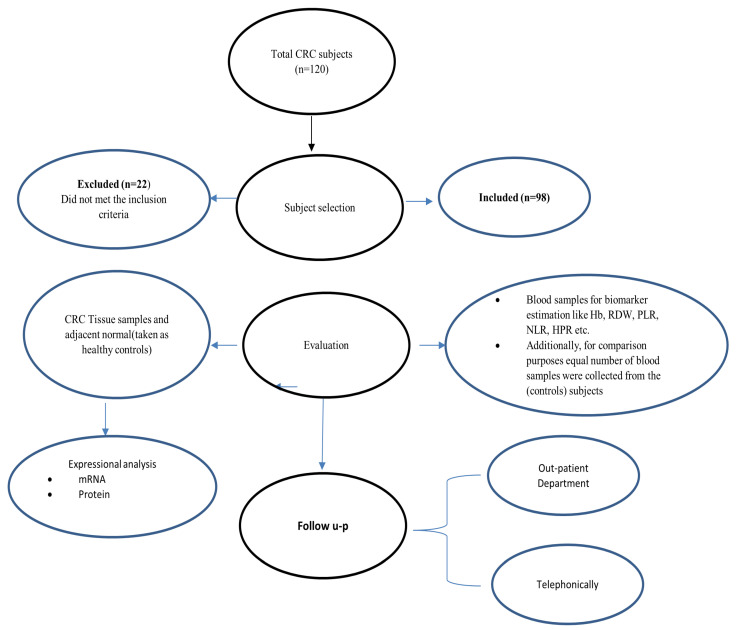
Flow diagram based on patient inclusion and exclusion criteria.

CRC patients treated with surgical resection with a histopathology-confirmed diagnosis were included in the study. Patients with anemia, hematological or systemic diseases, recent blood or platelet transfusions, and chemo- or radiotherapy were excluded, as shown in Figure 1. 

### 2.4. RNA Isolation, and cDNA Synthesis and Real-Time PCR of SuFu

To gain a better understanding of the clinical importance of *SuFu* expression in colorectal cancer, we evaluated *SuFu* expression using a number of clinicopathological and laboratory parameters. RNA was extracted using the Trizol method (Invitrogen Waltham, MA). For RNA, absorbance at A260/280 of 1.9–2 was considered “pure”. We used the RevertAid First Strand cDNA Synthesis Kit (Thermo Scientific Ltd., Waltham, MA, USA, #K1622) to synthesize complementary DNA (cDNA) after DNase-I (Qiagen) treatment. The reverse transcription of 1 g RNA was performed in a volume of 20 ul using AMV Reverse Transcriptase and random hexamers. The cycle’s thermal settings in this experiment were 5 min at 25 °C, 60 min at 42 °C, and lastly, 5 min at 70 °C. On a PikoReal PCR system (Thermo), quantitative real-time PCR (qRT-PCR) was performed using SYBR Green Master Mix (Thermo Fisher Scientific Ltd., Waltham, MA, USA). Each result was standardized to the housekeeping gene *GAPDH*, and the experiments were carried out in triplicate. The primers used were as follows: *SuFu*—F: 5-CGGAGGGGAGAGACCATATT-3, R: 5-CACTTGGCACTGACACCACT-3; *GAPDH*—F: 5-CACTTGGCACTGACACCACT-3′, R: 5-CTTCACCACCTTCTTGATG-3. For *SuFu* mRNA expression, the cycle threshold (Ct) was used. Based on Livak and Schmittgen’s 2^−∆∆ct^ method, the relative expression levels were determined. A qRT-PCR reaction mixture was incubated at 95 °C for 3 min, proceeded by 40 cycles of denaturation (15 s at 95 °C), annealing (20 s at 57 °C), and extension (20 s at 72 °C). According to the melt curve study, no non-specific products were generated.

### 2.5. Protein Expression and Localization of SuFu via Immunohistochemistry (IHC)

The protein expression and localization of *SuFu* via IHC was conducted in the Department of Pathology, SKIMS.

### 2.6. Protocol for IHC

Paraffin blocks were sectioned into 5 m thick tissue sections, and those sections were then mounted on charged poly-L-lysin-coated glass slides (Bio-Optica Milano S.p.a via San Faustino, 58 20134 Milan, Italy LOT #180310). Following deparaffinization in xylene, the slides were rehydrated with ethanol in a graduated sequence of concentrations (100 percent, 95 percent, 90 percent, 80 percent, and 70 percent), followed by distilled water. The sections were covered with hydrogen peroxide (Biocare Medical, Pacheco, CA, USA) to prevent peroxidase activity from occurring naturally, and then, incubated in a humid atmosphere for 15 min. Phosphate-buffered saline (PBS) (pH = 7.4) was then used to wash the sections two to three times. In order to recover antigens, the slides were heated to 95 degrees Celsius in 10 mM citrate buffer (pH 6.0). Then, PBST was used to wash the slides. After that, a protein block (Biocare Medical, USA #BS966G) was added to stop any background tissue staining that was not specific for 15 min. The portions were then washed using PBST (1× PBS with Tween 20). To indicate the borders of the tissue sections, a PAP pen was used to properly dry the slides without disrupting the tissue sections (Abcam, Cambridge, UK). Next, entire sections were incubated with anti-*SuFu* (HPA008700; 1:200) primary antibody overnight at 4 °C before being washed in PBST the following day. The samples were incubated for 30 to 40 min with goat anti-rabbit secondary antibody that had been HRP-conjugated (MACH 2 Universal HRP-Polymer Detection Kit; Catalog no. #M2U522G). PBST was applied to the sections two to three times. The HRP/DAB Detection IHC kit was used to color the sections (cat. no. BDB2004H; Biocare). The sections were once more PBST-washed three times. Following immunoreactivity, the slides were submerged in distilled water and counterstained with hematoxylin. The sections were then dehydrated in xylene and alcohol and mounted with DPX, and the cover was slipped. A light microscope (181; Olympus, 1 81 Tokyo, Japan) was used to view the slides. In the negative controls, primary antibody was not added to the tissue sections, and phosphate-buffered saline (PBS) was used.

### 2.7. Evaluation of IHC

The evaluation of the IHC slides and images was undertaken by two expert pathologists independently. The intensity of staining was measured using the IHC Profiler plugin for ImageJ software. Comparing adjacent histological normal slides to the tumor slides, staining intensities were determined. The staining intensity was designated as 0 (negative), 1 (weak), 2 (moderate), or 3 (high) and the percentage proportion of cells stained as 0 (0%), 1 (25%), 2 (26–50%), 3 (51–75%), or 4 (>75%). A scoring system (IRS = immunoreactive score) [40] with some modification was used to evaluate the IHC slides. This was achieved by multiplying the staining intensity by the percentage proportion of cells stained. An optimal cut-off score was identified and accordingly set for high and low expression of *SuFu* in CRC tumors cells. An IRS ≤ 4 was defined as tumors having low *SuFu* expression. 

### 2.8. Follow-Up

A follow-up examination was undertaken once every three months during the first two years following surgery, and once every six months until 3 years after surgery. Patients were followed up in the outpatient department (OPD) or by phone. The patient communication deadline was 2 April 2022. We estimated survival intervals based on both diagnosis and surgery dates.

### 2.9. Ethics

Before surgery, all patients were informed about the study, which was authorized by the SKIMS Ethical Clearance Committee under protocol no: RP 70/2019.

### 2.10. Statistical Analysis

SPSS (v.26), Graph Pad Prism 8, and MedCalc were used for statistical analysis. The Shapiro–Wilk normality test was used to ensure that the data were distributed normally. Continuous variables with normal distributions were reported as means ± standard deviation (SD), and differences between them were evaluated using the Student’s t-test. Mann–Whitney U tests were used to compare groups of data that did not fit the normal distribution. For categorical variables, the Chi-square test was applied. Spearman’s correlation was used to examine the relationship between two continuous variables that contradicted the normal distribution assumption, and the Kruskal–Wallis one-way ANOVA test was employed to examine group differences between more than two groups. Receiver-operating characteristic (ROC) curves were utilized to determine the diagnostic value of RDW, PLR, NLR, and HPR, and the Youden index was used to establish the appropriate cut-off value for all of them to distinguish between CRC patients and healthy controls. To compare survival rates between groups, the Kaplan–Meier method and the log-rank test were utilized. For multivariate analysis, the Cox proportional hazard model was used. All tests were two-tailed, with a *p*-value of <0.05 considered statistically significant.

## 3. Results

### 3.1. Patient Characteristics

The expression of *SuFu* was assessed in 98 histopathologically validated tissues and adjoining normal tissues that had not received chemo or radiotherapy. Table 1 presents the study population’s demographic and clinicopathological characteristics. The patients included 57 (58.16%) males and 41 (41.83%) females. The mean age was 57.51 ± 13.9, and 29 (29.59%) were younger than the age of 50 years, whereas 69 (70.40%) patients were older than or equal to 50 years. Among all the subjects, 69 (70.40%) lived in rural areas and 29 (29.59%) lived in urban areas.

### 3.2. SuFu mRNA Expression in CRC

**Figure 2:**
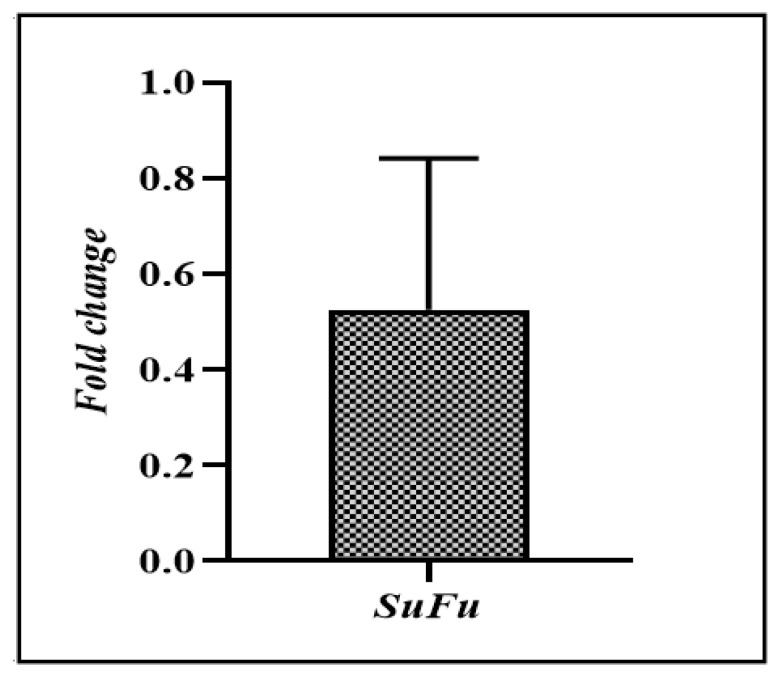
Average fold change in *SuFu* in CRC malignant tumor tissues.

Overall, low expression was seen in 69 (70.40%) of the malignant CRC tumor tissues relative to the adjacent normal tissues. As shown in Figure 2, the average fold change in SuFu was 0.550 ± 0.44.

**Figure 3:**
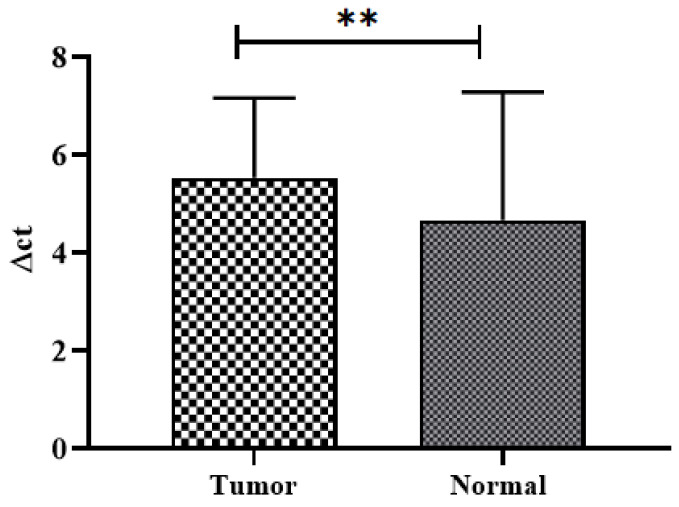
Graph illustrating the relative mRNA expression of *SuFu* in CRC patients by comparing ∆ct values of tumor and adjacent normal tissues. mRNA: messenger RNA; qRT-PCR: quantitative real-time polymerase chain reaction; ** denotes significance.

When comparing the relative mRNA expression of tumor and adjacent normal tissues in terms of ∆ct values, overall, malignant tumors displayed reduced expression, compared to adjacent normal tissues, as shown in Figure 3.

### 3.3. Comparison of SuFu mRNA Expression with Various Clinicopathological and Laboratory Parameters

To gain a better understanding of the clinical importance of *SuFu* expression in colorectal cancer, we evaluated *SuFu* expression using a number of clinicopathological and laboratory parameters. The expression of *SuFu* was significantly associated with gender, education, passive smoking, stage, node status, and recurrence (all parameters have *p* < 0.05). There was no association with age, blood group, social class, junk food, tumor depth, tumor site, tumor size, and various other parameters listed in Table 2.

### 3.4. SuFu Protein Expression and Localization via IHC

**Figure 4:**
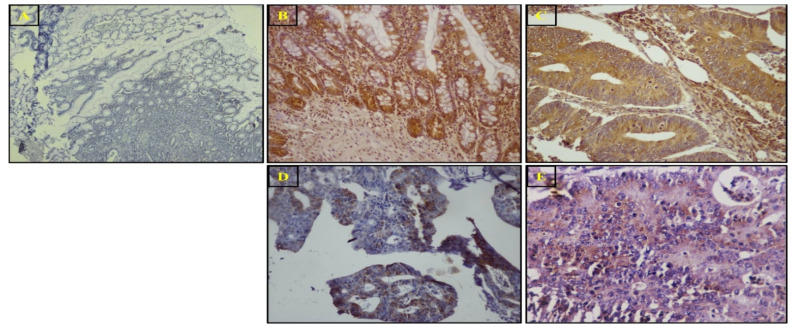
Representative immunohistochemical images showing expression of the *SuFu* protein in colorectal cancer and adjacent normal tissues: (**A**) negative control; (**B**) strong staining in normal tissue; (**C**) strong nucleo-cytoplasmic staining in low-grade tumor (grade 1: well differentiated); (**D**) moderate nucleo-cytoplasmic staining (grade 2: moderately differentiated); (**E**) weak cytoplasmic staining in high-grade CRC tumor (grade 3: poorly differentiated).

**Figure 5:**
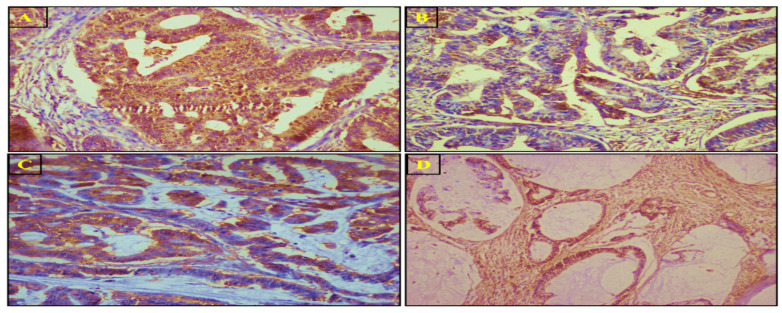
Representative immunohistochemical images showing expression of the *SuFu* protein in colorectal cancer tumors: (**A**) nucleo-cytoplasmic; (**B**) nuclear; (**C**) cytoplasmic staining; (**D**) mucinous (nucleo-cytoplasmic).

Immunohistochemistry was used to evaluate the expression and localization of the *SuFu* protein. *SuFu* was downregulated in a higher number of tumor samples (*n* = 62, 63.2%) than adjacent normal tissues and was predominantly localized in the nucleo-cytoplasm, followed by the nucleus and the cytoplasm. The staining was mostly moderate-to-strong in normal adjacent samples. *SuFu* was less expressed and downregulated in high-grade tumors. Figure 4 represents the staining pattern of *SuFu* in CRC tumors and adjacent normal tissues. The nucleo-cytoplasmic, nuclear, cytoplasmic, and mucinous staining patterns for *SuFu* are illustrated in Figure 5. *SuFu* expression was significantly correlated with tumor differentiation (grade), tumor invasion depth, stage, PNI, LNM, node status, recurrence, and vital status. The correlation was statistically significant (all parameters had *p*-value *<* 0.05). However, there was no association between *SuFu* expression and other parameters such as tumor site, tumor size, and other variables, as indicated in Table 3.

Further investigation was carried out to determine whether the reduced *SuFu* expression at the transcriptional and translational levels are related. On comparison, we found that 53 (82.81%) of the malignant tumor tissues that displayed reduced expression at the mRNA level also showed decreased *SuFu* expression at the protein level. The change was significant. Tumor tissues that displayed low mRNA *SuFu* expression also expressed low *SuFu* protein (*p* < 0.001), as presented in Table 4. 

### 3.5. Comparison of Laboratory Parameters between CRC Group and Healthy Controls

The median serum level of CEA and CA-19.9 in CRC patients was 5.60 (*IQR*: 2.14–17.36) and 20.35 (*IQR*: 8.95–46.08). The comparison of the two groups is shown in Table 5. The neutrophil, RDW, PLR, NLR, and PLT values were significantly higher in the CRC group than in the healthy controls. The difference was statistically significant (for all parameters, *p* < 0.05). However, HB, HPR, and lymphocytes were lower in the CRC group compared to the healthy controls. The difference observed was significant (*p* < 0.05 for all). However, there was no difference in age and WBC count.

### 3.6. Correlation of Laboratory Parameters with Different Clinicopathological Parameters in Patients with CRC

This relationship is summarized in Table 6. RDW showed a significant difference with tumor site, necrosis, node status, perineural invasion (PNI), tumor depth, and stage (*p* < 0.05 for all). HPR showed a significant difference with necrosis, tumor depth, stage, lymphovascular invasion (LVI), tumor size, and CEA (*p* < 0.05 for all). PLR showed a significant difference with necrosis and distant metastasis (*p* < 0.05 for both). NLR showed a significant difference with tumor-associated lymph node response (TALNR) and tumor site (*p* < 0.05 for both).

### 3.7. Diagnostic Efficacy of RDW, PLR, NLR, and HPR, Used Alone or in Combination, in Differentiating Colon Cancer from the Normal Healthy Control Group

**Figure 6:**
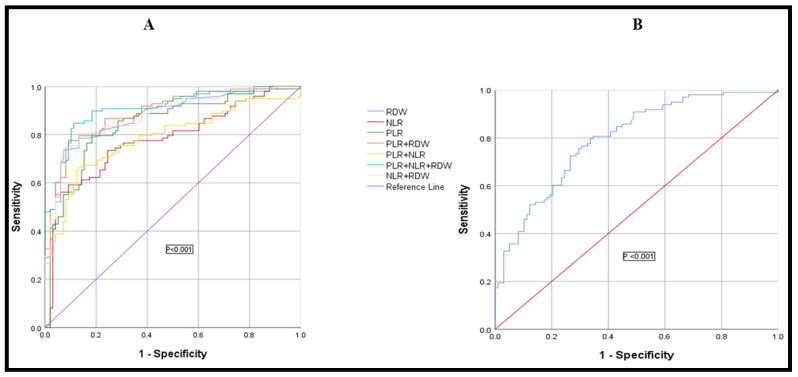
(**A**) Analysis of receiver operating characteristic curves for RDW, NLR, and PLR biomarkers, used alone or together, for differentiating colon cancer patients from healthy controls. (**B**) Analysis of receiver operating characteristic curve for HPR biomarker used alone for differentiating colon cancer patients from healthy controls. NLR: neutrophil-to-lymphocyte ratio; PLR: Platelet-to-lymphocyte ratio; HPR: hemoglobin-to-platelet ratio; RDW: red cell distribution width.

When differentiating colon cancer from a healthy control group, the laboratory parameters RDW, PLR, NLR, and HPR were assessed for sensitivity, specificity, positive and negative likelihood ratios, and area under the receiver operating characteristic curves (AUC). Youden’s index set the cut-off value. Table 7 and Figure 6A show that RDW, PLR, NLR, and HPR had a sensitivity of 73.5%, 79.6%, 59.18%, and 72.45%, respectively, in discriminating colon cancer from healthy controls. RDW had the highest specificity (92.9%). When PLR, NLR, and RDW were combined, the AUC (0.91; 95% CI: 0.86–0.94) was higher than when PLR (AUC = 0.84), NLR (AUC = 0.78), RDW (AUC = 0.87), and HPR (AUC = 0.79), (Figure 6B) were utilized alone.

### 3.8. Correlations between Laboratory Parameters in CRC Patients

**Figure 7:**
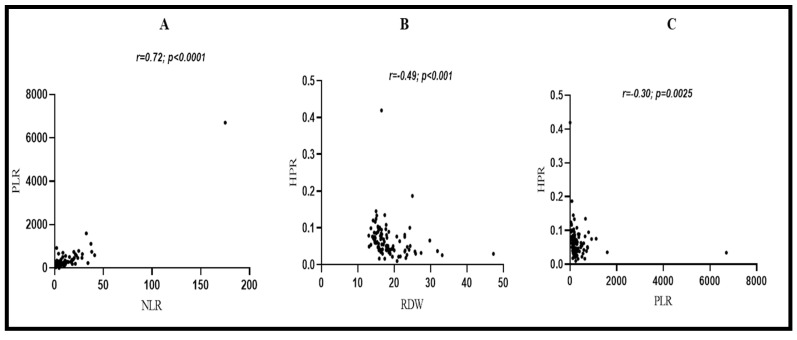
(**A**) Strong positive correlation between PLR and NLR (*p* < 0001); (**B**) moderate negative correlation between HPR and RDW (*p* < 0.001); (**C**) weak negative correlation between HPR and PLR. HPR: hemoglobin-to-platelet ratio; RDW: red cell distribution width; PLR: platelet-to-lymphocyte ratio.

Figure 7A–C, depicts the relationship between PLR and NLR, HPR and RDW, and HPR and PLR. In CRC patients, there was a strong positive correlation between PLR and NLR (r = 0.72, *p* < 0.001). HPR showed a moderate negative correlation with RDW (r = −0.49, *p* < 0.001), and a weak negative correlation was found between HPR and PLR (r = −0.30, *p* < 0.001).

### 3.9. HPR Value’s Association with Cancer Stage and Tumor Invasion Depth

**Figure 8:**
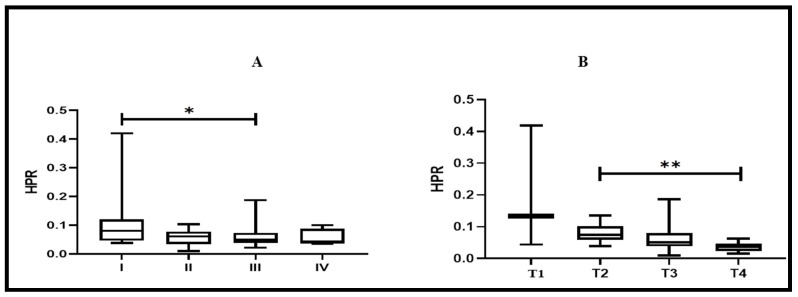
(**A**) Association of HPR with tumor stage (I vs. III, *p* < 0.012) in CRC; (**B**) association of HPR with tumor depth (T2 vs. T4, *p* < 0.021). HPR: hemoglobin-to-platelet ratio; CRC: colorectal cancer; HPR: hemoglobin-to-platelet ratio; * denotes significance; ** denotes significance.

To compare HPR values among different cancer stages and tumor invasion depths, a Kruskal–Wallis one-way Analysis of Variance (ANOVA) was employed. As shown in Figure 8A,B, there was a significant association of HPR with stages I and III (*p* < 0.012) and with T2 and T4 (*p* < 0.021).

### 3.10. Prognostic Analysis of Various Clinicopathological Parameters

**Figure 9:**
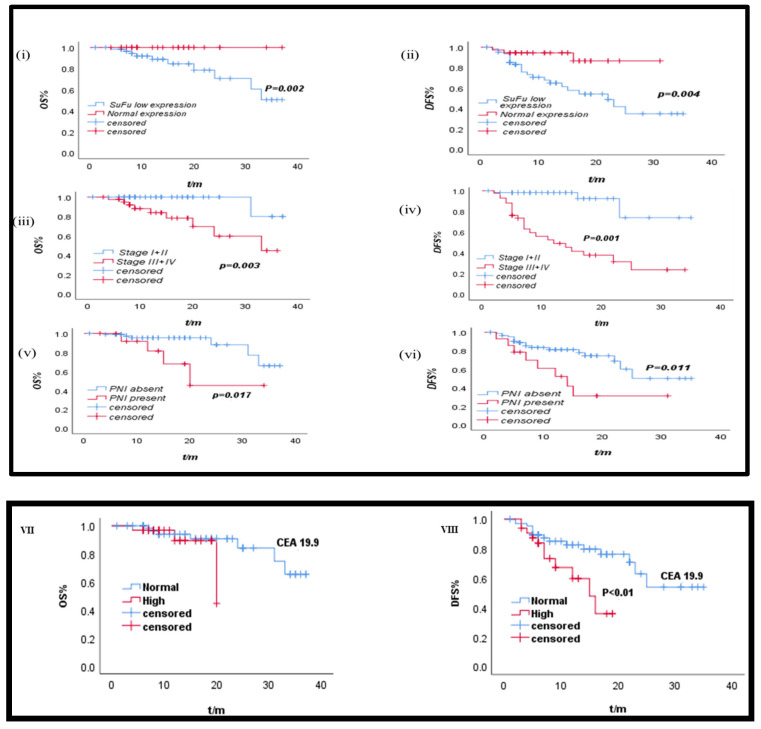
Kaplan–Meier survival probability curves. (**i**,**ii**): *SuFu* Expression with OS and DFS; (**iii**,**iv**): stage I + II and III + IV with OS and DFS; (**v**,**vi**): PNI with OS and DFS; (**VII**,**VIII**): CEA 19.9 with OS and DFS; PNI: perineural invasion; OS: overall survival; DFS: disease-free survival.

A number of pathological parameters were assessed for prognostic significance in CRC by conducting univariate survival analysis with the 3-year disease-free survival (DFS) and overall survival (OS) rates of the patient cohort (Table 8). Stage, PNI, node status, LNM, and *SuFu* expression were all significantly linked with both 3-year OS and 3-year DFS. However, CA 19-9 exhibited a significant correlation with DFS rate (*p* < 0.05). The prognosis was not significantly influenced by the tumor grade, tumor depth, lymphovascular invasion (LVI), or tumor site. Figure 9, depicts Kaplan–Meir survival curves, based on IHC for 3-year OS and DFS dependent on *SuFu* expression, stage, and PNI. Lower *suFu* expression, PNI positivity, and presence of positive axillary nodes were all associated with the worst OS and DFS rates (*p* < 0.05). Based on Cox regression analysis (Table 9), a significantly independent predictive association was observed between CA-19.9 protein and 3-year DFS (*p* < 0.05).

Finally, we correlated various lab inflammatory blood biomarkers with *SuFu* expression (Table 10). Although RDW, PLR, and NLR values were higher in most of the patients who displayed low *SuFu* expression, the association was not statistically significant. The HPR values were decreased in most of the patients exhibiting low *SuFu* expression, but the findings were not significant.

## 4. Discussion

In the present study, *SuFu* mRNA and protein expression were evaluated in histopathologically confirmed tumor tissues and adjacent normal tissues. The findings show that *SuFu* is downregulated in CRC tumor tissues at both the mRNA and protein levels with predominant nucleo-cytoplasmic localization. Patients with low *SuFu* expression exhibited poor prognosis. Further high-grade tumors exhibited significantly lower *SuFu* expression than low-grade tumors. 

We found that 70.4% of CRC patients had lower *SuFu* mRNA expression, which is comparable to previous observations by wang et al. [41] We, for the first time, evaluated *SuFu* expression in relation to a number of demographic and clinicopathological variables and found that low *SuFu* expression is associated with late stage, the presence of positive axillary lymph nodes, lower education level, recurrence, female sex, and passive smoking. An earlier study demonstrated that *SuFu* expression is correlated with tumor invasion depth and tumor diameter [41]. Education is believed to be inversely related to cancer risk [42,43]. Even though a lower education level does not cause cancer at the molecular level, it may impact the risk through behavior, standard of living, and environmental exposure. However, in the current study, a high proportion of participants were from the lower education group. Even though the correlation is statistically significant, it may be coincidental. More studies with larger sample sizes could provide a more accurate picture. Passive smokers exhibited a significantly low expression of *SuFu*. A previous study conducted on our population by Rafiq et al. [44] linked second-hand smoking with esophageal cancer risk. Moreover, abnormal expression in females, especially in rural areas, could be attributed to poor house design leading to inadequate ventilation, which exposes them to different pollutants such as cooking fumes [45].

IHC analysis revealed that *SuFu* was downregulated in 63.3% of the malignant tumor tissues relative to adjacent normal tissue samples. The majority of the malignant tumor samples displayed nucleo-cytoplasmic localization of *SuFu*, followed by the nucleus and the cytoplasm. In a previous study by Tostar et al., *SuFu* was found to be weakly expressed and predominant in the cytoplasm of tumor muscle cells [46]. *SuFu* was expressed in normal colon tissues. This is evident, because in species ranging from invertebrates to vertebrates, it plays a crucial part in embryogenesis and adult tissue homeostasis. *SuFu* has vital functions in mammals, as evidenced by the embryonic lethality of *SuFu* deletion in mice [47]. However, our results are not in agreement with the findings of Wang et.al, who found no *SuFu* in normal colonic mucosa in their study [41]. Lower levels of *SuFu* protein were linked to high-grade tumors, tumor invasion, late stage, node presence, LNM positivity, PNI positivity, CRC recurrence, and vital status. Our findings indicate that *SuFu* is downregulated and weakly expressed in high-grade tumors in CRC relative to lower-grade tumors. This is the first study that reports the downregulation of *SuFu* along higher grades in CRC tumors. Interestingly, just one study with a very small sample size reported similar findings in tumors originating from glioblastoma [48]. Based on our results, *SuFu* has promising prognostic implications. Moreover, the association between low *SuFu* expression and LNM, PNI, and late-stage CRC suggests that *SuFu* is associated with the worst pathological features and advanced stages of the disease. 

We also found that a low *SuFu* expression, in addition to shorter and worse overall survival, is associated with poor disease-free survival. Positive axillary lymph nodes, PNI, and LNM showed similar relationships with OS and DFS. Similarly, patients in stage III and IV demonstrated the shortest and worst overall survival and disease-free survival rates. According to our findings, in addition to stage, low *SuFu* expression, PNI, and LNM are connected to CRC metastases and have a possible prognostic role. According to the Cox regression analysis, the best predictor for DFS was preoperative CA-19.9 levels (RR: 3.7, 95% CI: 1.32–7.11, *p* < 0.05). Based on these findings, we can say that preoperative CA-19-9 has a significant role in predicting the prognosis for colon cancer patients receiving surgery.

In this study, we found that RDW values were significantly increased in advanced tumor invasion depth (T3 and T4), later stages (III and IV), PNI, necrosis, node status, and tumor site (*p* < 0.05 for each parameter). No association was found between RDW values and lymph node metastases (LNM), tumor configuration, and tumor size. An earlier study conducted by Shi et.al, reported that elevated RDW is associated with tumor type, tumor invasion depth (T status), clinical stage, and histological type [49]. However, our study did not find any significant differences in RDW values in tumors of the colon or rectum. However, our study is the first study to report that right colon tumors are associated with higher RDW values than left colon tumors. To the best of our knowledge, this is also the first study that has revealed the association of RDW with PNI and necrosis in CRC. Similarly, Elevated PLR was found in DM and necrosis (*p* < 0.05 for both). A previous study conducted by Hu et al. reported elevated PLR in DM [23], but Mo et al. did not find any association between PLR and DM [50]. A significant association between elevated PLR and necrosis was also noted. This is the first study to reveal the link between necrosis and elevated PLR in CRC. However, the underlying mechanism is still largely unknown. Additionally, earlier studies did not include many clinicopathological data. Further, for the first time, our study also reported elevated NLR in tumors of the transverse colon and in patients where tumor-associated lymph node response (TALNR) was seen (*p* < 0.05 for both). Additionally, lower HPR values were associated with tumor stage (III and IV), tumor invasion depth (T3 and T4), LVI, necrosis, tumor size (≥4 cm), and high CEA level (*p* < 0.05 for all). These findings are consistent with the results given by Hu et al. and Mo et al. in their previously conducted studies [23,50].

Further, the diagnostic efficacy of RDW was excellent (AUC = 0.87) (*p* < 0.05), and was much higher than that reported by Song et al. [21] and Shi et al. [49] in separate studies, suggesting the greater utility of RDW as a diagnostic parameter in our population. The diagnostic value of PLR (AUC = 0.84) and HPR (AUC = 0.79) was also higher than in studies conducted by Mo et al. [50] and Hu et al. [23], respectively. To the best of our understanding, NLR has not been used or tested for diagnostic purposes so far. In our study, NLR yielded moderate diagnostic utility (AUC = 0.78) (*p* < 0.05). However, upon combining RDW, NLR, and PLR, the diagnostic performance was found to be excellent, and a larger area under the curve was obtained (AUC = 0.91, 95% CI: 0.86–0.93, *p* < 0.05). Our results suggest that the combined use of RDW, NLR, and PLR may greatly improve the diagnostic efficacy of differentiating CRC from normal healthy controls compared to using them alone. Further, in CRC patients, we conducted a correlation analysis for RDW, NLR, PLR, and HPR. We found a strong positive correlation between PLR and NLR (r = 0.72, *p* < 0.05), a moderate negative correlation between RDW and HPR (r = −0.49, *p* < 0.05), and a weak negative correlation between HPR and PLR (r = −0.30, *p* < 0.05). The exact mechanism for such a relationship is still not clear and no studies that reported such findings are available yet. We further stratified CRC subjects based on stage (I, II, III, and IV) and tumor invasion depth (T1, T2, T3, and T4). Interestingly, we found that HPR was significantly decreased in stage III CRC patients and in advanced tumor invasion depth (T4), when compared with stage I and tumor invasion depth (T2) (*p* < 0.05 for both).

Lastly, we evaluated whether the mRNA expression of *SuFu* at the genetic level would affect RDW, PLR, NLR, HPR, and CEA levels. We wanted to identify whether abnormal parameter values and low expression were interconnected. A relationship between them was not established. Both low expression and altered levels of these parameters may play a role in CRC pathogenesis independently. It is nevertheless possible for this study to serve as a platform for future investigations, guiding them toward a combination approach at both the expressional and clinical levels.

Unlike the previous studies mentioned above [50,51,52,53,54], our study was a prospective one. In addition, both blood and tissue samples were included. A proper follow-up procedure was followed. Due to time and resource constraints, we were unable to ascertain the MSS/MSI status of tumors, which was the study’s principal shortcoming. This study’s survival proportion was affected by the fact that it lasted for only three years.

## 5. Conclusions

*SuFu* was downregulated in CRC tumor tissues at both the mRNA and protein levels. Pathological features such as high-grade tumor, nodes, PNI, and LNM were associated with low *SuFu* expression. The results of our study indicate that in addition to stage, low *SuFu* expression, PNI, and LNM have excellent prognostic value. *SuFu* may be a potential therapeutic target in CRC. Furthermore, RDW, PLR, NLR, and HPR were associated with a range of clinicopathological variables, several of which had not previously been reported. Using RDW, PLR, and NLR together may provide excellent diagnostic ability to differentiate CRC from healthy control groups. In patients with colon cancer who are undergoing surgery, the preoperative CA-19-9 level can be a significant predictor of disease-free survival.

## Figures and Tables

**Table 1:** General characteristics of the study population (*n* = 98).

Characteristics	Number (n) & Percentage (%)
**Age**	
<50	29(29.6)
≥50	69(70.4)
**Gender**	
Male	57(58.2)
Female	41(41.8)
**Dwelling**	
Rural	69(70.4)
Urban	29(29.6)
**Social Class**	
Low	40(40.8)
Middle & High	58(59.2)
**Education**	
lower	65(65.3)
higher	33(33.7)
**Blood Group**	
A	23(23.5)
B	33(33.7)
AB	18(18.3)
O	24(24.5)
A+B	56(57.14)
AB+O	42(42.90)
**BMI**	
<24.9	59(60.2)
25–29.9	31(31.6)
≥30	8(8.2)
<25	59(60.2)
≥25	39(39.8)
**Family History**	
Yes	24(24.5)
No	74(75.5)
**Smoking Status**	
Yes	38(38.8)
No	60(61.2)
**Lifestyle**	
Active	68(69.4)
Sedentary	30(30.6)
**Comorbid Status**	
Present	40(40.8)
Absent	58(59.2)
HTN	24(24.5)
HTN+T2D	16(16.3)
Absent	58(59.2)
**Salt Intake**	
Yes	82(83.7)
No	16(16.3)
**Red Meat Consumption**	
Yes	74(75.5)
No	24(24.5)
**Sundried Vegetables**	
Yes	76(77.6)
No	22(22.4)
**Source of Drinking Water**	56(57.2)
Tap Water (R)	26(26.5)
Tap Water (L)	16(16.3)
Others	
**Pickles**	
Yes	73(74.5)
No	25(25.5)
**Pesticide Exposure**	
Yes	31(31.6)
No	67(68.4)
**Junk Food Consumption**	
Yes	16(16.3)
No	82(83.7)
**Frying**	
Shallow	57(58.2)
Deep	41(41.8)
**Histological type**	
Adenocarcinoma	88(89.8)
Mucinous	10(10.2)
**Site of Tumor**	
Colon(C)	56(57.1)
Rectum(R)	29(29.6)
Rectosigmoid(RS)	13(13.3)
RC	24(24.5)
TC	8(8.1)
LC	24(24.5)
RS	13(13.3)
R	29(29.6)
Colon	69(70.4)
Rectum	29(29.6)
**Tumor configuration**	
Ulcerated	27(27.6)
Ulceroinfilitrative	71(72.4)
**Tumor size (cm)**	
1–3	38(38.8)
≥3	60(61.2)
**Tumor Differentiation**	
Well	15(15.3)
Moderate	69(70.4)
Poor	14(14.3)
**Tumor Invasion Depth**	
T1	3(3.1)
T2	22(22.5)
T3	61(62.2)
T4	12(12.2)
T1 + T2	25(25.5)
T3 + T4	73(74.5)
**TNM Staging**	
I	17(17.3)
II	39(39.8)
III	38(38.8)
IV	4(4.1)
I + II	56(57.1)
III + IV	42(42.9)
**Tumor Grade**	
1	15(15.3)
2	69(70.4)
3	14(14.3)
**Node Status**	
Absent	57(58.2)
Present	41(41.8)
**Necrosis**	
Present	29(29.6)
Absent	69(70.4)
**LVI**	
Present	66(67.3)
Absent	32(32.7)
**PNI**	
Present	14(14.3)
Absent	84(85.7)
**Distant Metastasis**	
Present	3(3.1)
Absent	95(96.9)
**TALNR**	
Present	62(63.3)
Absent	36(36.7)
Poor	12(12.3)
Mild-moderate	39(39.8)
High	11(11.22)
**Necrosis**	
Yes	29(29.6)
No	69(70.4)
**Recurrence**	
Yes	26(26.5)
No	72(73.5)
**Vital Status**	
Alive	88(89.8)
Dead	10(10.2)

BMI: body mass index; HTN: hypertension; T2D: Type II diabetes; PNI: perineural invasion; LVI: lymphovascular invasion; TALNR: tumor-associated lymph node response; RC: right colon; TC: transverse colon; LC: left colon; RS: rectosigmoid; T1: tumor invades mucosa and submucosa; T2: tumor invades muscularis propria; T3: tumor invades subserosa; T4: tumor invades serosa.

**Table 2:** Comparison of *SuFu* expression with different demographic and clinicopathological variables in study population.

Characteristics	Low Expression *n* (%)	Same as Normal *n* (%)	OR (95%CI)	*Chi2*	*p*-Value
**Age**					
≤50	22(75.8)	7(24.2)	1.47(0.54–3.95)	0.44	0.581
>50	47(68.1)	22(31.9)			
**Gender**					
Male	35(61.4)	22(38.6)	0.32 (0.124–0.866)	5.32	**0.021**
Female	34(83)	7(17)			
**Dwelling**					
Rural	48(69.5)	21(30.4)	0.87(0.33–2.27)	0.80	0.781
Urban	21(72.4)	8(27.6)			
**Social Class**					
Low	29(72.5)	11(27.5)	1.18(0.48–2.88)	1.42	0.712
Middle & High	40(70)	18(30)			
**Education**					
lower	52(80)	13(20)	3.7(1.52–9.31)	8.52	**0.004**
higher	17(51.5)	16(48.5)			
**Blood Group**					
A+B	42(75)	14(25)	1.6(0.69–3.99)	1.32	0.250
AB+O	27(64.3)	15(35.7)			
**BMI**					
<25	43(72.9)	16(27.1)	1.3(0.55–3.23)	0.42	0.509
≥25	26(66.7)	13(33.3)			
**Family History**					
Yes	16(66.7)	8(33.3)	1.2(0.47—3.38)	2.14	0.64
No	53(71.6)	21(28.4)			
**Comorbid Status**					
Present	27(67.5)	13(32.5)	1.2(0.52–3.03)	0.271	0.621
Absent	42(72.4)	16(27.6)			
**Smoking Status**					
Yes	20(52.6)	18(47.4)	1.1(0.91–9.98)	9.41	0.058
No	49(81.7)	11(18.3)			
**Passive smoking**					
Yes	58(82.8)	12(17.2)	0.13(0.051–0.351)	18.2	**0.001**
No	11(39.3)	17(60.7)			
**Lifestyle**					
Active	51(75)	17(25)	0.50(0.20–1.2)	2.24	0.134
Sedentary	18(60)	12(40)			
**Source of Drinking Water**					
Tap (River+Lake)	55(67.1)	27(32.9)	0.29(0.062–1.37)	2.6	0.102
Other	14(87.5)	2(12.5)			
**Frying**					
Shallow	38(66.7)	19(33.3)	0.64(0.26–1.58)	0.913	0.339
Deep	31(75.6)	10(24.4)			
**Pesticide Exposure**					
Yes	19(61.3)	12(38.7)	1.8(0.749–4.60)	1.80	0.179
No	50(74.6)	17(25.4)			
**Junk Food Consumption**					
Yes	10(62.5)	6(37.5)	1.5(0.502–4.72)	0.57	0.449
No	59(72)	23(28)			
**Histological type**					
Adenocarcinoma	62(70.4)	26(29.6)	0.97(0.23–4.08)	0.01	0.976
Mucinous	7(70)	3(30)			
**Site of Tumour**					
Colon	38(67.8)	18(32.2)	0.3(0.07–1.91)	1.46	0.481
Recto sigmoid	11(84.6)	2(15.4)			
Rectum	20(69)	9(31)			
**Tumor configuration**					
Ulcerated	19(70)	8(30)	0.98(0.37–2.63)	0.088	0.996
Ulceroinfilitrative	50(70.4)	21(29.6)			
**Tumour Differentiation**					
Well	12(80)	3(20)	2.1(0.5–8.3)	3.12	0.209
Moderate	45(65.2)	24(34.8)			
Poor	12(85.7)	2(14.3)			
**Tumor size**					
<3cm	28(66.7)	14(33.3)	0.73(0.30–17)	4.94	0.482
≥3cm	41(73.2)	15(26.8)			
**Tumor Invasion Depth**					
T1 + T2	14(56)	11(44)	0.41(0.16–1.08)	3.34	0.067
T3 + T4	55(75.3)	18(24.7)			
**TNM Staging**					
I	7(41.2)	10(58.8)	2(0.08–0.90) - 0.39(0.15–0.91)	10.01 3.92	**0.018** **0.048**
II	28(71.8)	11(28.2)			
III	30(79)	8(21)			
IV	4(100)	0(0)			
I + II	35(62.5)	21(37.5)			
III + IV	34(81)	8(19)			
**Necrosis**					
Present	22(76)	7(24)	0.68(0.25–1.82)	0.58	0.443
Absent	47(68)	22(32)			
**Node status**					
Present	34(83)	7(17)	0.32(0.12–0.86)	5.32	**0.021**
Absent	35(61)	22(39)			
**LVI**					
Present	48(72.7)	18(27.3)	0.71(0.28–1.77)	0.52	0.470
Absent	21(65.5)	11(34.5)			
**PNI**					
Present	10(71.4)	4(28.6)	0.94(0.27–3.29)	0.08	0.928
Absent	59(70.2)	25(29.8)			
**Distant Metastasis**					
Present	3(100)	0(0)	–	1.30	0.254
Absent	66(69.4)	29(30.6)			
**TALNR**					
Present	45(72.5)	17(27.5)	0.75(0.31–1.83)	0.38	0.536
Absent	24(52.1)	22(47.9)			
**Recurrence**					
Yes	23(88.5)	3(11.5)	0.25(0.03–0.82)	5.53	**0.019**
No	46(63.9)	26(36.1)			
**Vital Status**					
Alive	64(72.7)	24(27.3)	0.21(0.04–0.98)	4.52	0.371
Dead	5(50)	5(50)			

**Table 3:** Comparison of *SuFu* IHC expression with various pathological features.

Characteristics	Low Expression (IRS ≤ 4)	Same Expression (IRS > 4)	OR (95%CI)	*Chi2*	*p*-Value
**Site of Tumour**					
Colon	35(65.5)	21(37.5)	0.91(0.2–2.6) 1.1(0.4–2.9)	0.94	0.925
Recto sigmoid	8(61.5)	5(38.5)			
Rectum	19(65.5)	10(34.5)			
**Tumor configuration**					
Ulcerated	16(59.3%)	11(40%)	0.7(0.3–1.9)	0.257	0.612
Ulceroinfilitrative	46(64.8%)	25(35%)			
**Tumour Differentiation (Grade)**					
Well(I)	5 (33.3%)	10(66.7%)	0.2(0.04–0.8) 0.2(0.08–0.9)	4.64 4.92	**0.042** **0.021**
Moderate(II)	27(39.4%)	42(60.7%)			
Poor(III)	10(71.4%)	4(28.6%)			
Tumor size					
<3 cm	15(78.9%)	4(21.1%)	2.5(0.7–8.4)	2.49	0.114
≥3 cm	47(59.5%)	32(40%)			
**Tumor Invasion Depth**					
T1 + T2	21(84%)	4(16%)	4(1.2–13.13)	6.2	**0.013**
T3 + T4	41(56.2%)	32(43.8%)			
**TNM Staging**					
I + II	27(48.2%)	29(51.8%)	3.1(1.5–6.3)	12.7	**0.003**
III + IV	35(83.3%)	7(16%)			
**Necrosis**					
Absent	44(71%)	25(69.4%)	1.02(0.7–1.3)	0.025	0.873
Present	18(29%)	11(30.6%)			
**Node status**					
Absent	28(49.1%)	29(50.9%)	2.9(1.4–6.1)	11.75	**0.001**
Present	34(82.9%)	7(17.1%)			
**LVI**					
Absent	16(50%)	16(50%)	0.4(0.1–1.03)	3.59	0.058
Present	46(69.7%)	20(30.3%)			
**LNM**					
Present	17(85%)	3(15%)	2.8(0.9–8.2)	5.1	**0.024**
Absent	45(57.7%)	33(42.3%)			
**PNI**					
Present	14(100%)	0(0%)	0.57(0.4–0.6)	9.4	**0.002**
Absent	48(57.1%)	36(42.9%)			
**Distant Metastasis**					
Present	3(100%)	0(0%)	0.6(0.5–0.7)	1.7	0.180
Absent	59(62.1%)	36(37.9%)			
TALNR					
Present	39(62.9%)	23(37.1%)	1.04(0.44–2.4)	0.010	0.922
Absent	23(63.9%)	13(36.1%)			
**Recurrence**					
No	39(54.2)	33(45.8)	3.9(1.3–11)	9.66	**0.002**
Yes	23(88.5)	3(11.5)			
**Vital Status**					
Dead	10 (100%)	0(0%)	1.6 (1.4–2.0)	6.4	**0.011**
Alive	52(59%)	36(40%)			

IRS: immuno-reactive score.

**Table 4:** Comparison of mRNA and protein expression of *SuFu* gene.

	Protein Expression			*Chi2*	*p*-Value
**mRNA expression**		Low expression	Same as normal	20.06	**0.0001**
	Low expression	53 (82.81%)	11 (17.19%)		
	Same as normal	13 (38.24%)	21 (61.76%)		

**Table 5:** Comparative analysis of CRC and control group.

Parameters	CRC Group	Healthy Controls	*p*-Value
	Mean/Median ± SD/IQR	Mean/Median ± SD/IQR	
**Age**	57.51 ± 13.9	55.16 ± 16.4	0.298
**HB (g/L)**	11.25 (10.10–12.30)	13.10 (11.9–14.6)	0.001
WBC (* 10^9^/L)	7.1 (5.60–9.12)	7.30 (5.13–8.76)	0.247
Neutrophils (* 10^9^/L)	4.95 (3.5,7.7)	4.01 (2.6–5.3)	0.001
Lymphocytes (* 10^9^/L)	1.10 (0.57–1.82)	1.96 (1.33–2.72)	0.001
PLT (* 10^9^/L)	186.50 (137.75–258)	129 (81.67–178.33)	0.001
**PLR**	187.85 (114.00–342.41)	65.10 (43.81–102.20)	0.001
**NLR**	4.93 (2.10–12.03)	1.93 (1.48–2.15)	0.001
**HPR**	0.055 (0.039–0.0811)	0.104 (0.073–0.1553)	0.001
**RDW%**	17.4 (15.57–20.72)	14.36 (13.48–15.09)	0.001

HB: hemoglobin; WBC: white blood cells; PLT: platelets; PLR: platelet-to-lymphocyte ratio; NLR: neutrophil-to-lymphocyte ratio; HPR: hemoglobin-to-platelet ratio; RDW: red cell distribution width; * denotes a multiplication sign.

**Table 6:** Association of clinicopathological variables with laboratory parameters in CRC patients.

RDW			
Clinicopathological Parameter	*N*	Median(IQR)	*p*-Value
**Tumor site**			
Right colon	24	21.25(18.12–24.25)	**0.001**
Left colon	24	17.40(15.20–18.50)	
**Tumor size**			
<3	38	17.20(15.18–20.03)	0.473
≥3	60	17.80(15.80–21.65)	
**Necrosis**			
Absent	69	16.60(15.40–19.65)	**0.025**
Present	29	18.50(16.80–22.90)	
**Tumor configuration**			
Ulcerated	27	16.80(15.20–20.02)	0.510
Ulceroinflitrative	71	17.80(15.80–21.65)	
**Node status**			
Absent	57	16.60(15.15–19.90)	**0.038**
Present	41	18.50(16.10–22.90)	
**LNM**			0.380
Absent	78	17.35(15.50–20.73)	
Present	20	18.60(16.45–20.90)	
**Tumor depth**			
T1 + T2	25	16.50(14.70–17.60)	**0.010**
T3 + T4	73	18.0(15.85–21.35)	
**Stage**			
I + II	56	16.70(15.12–19.47)	**0.026**
III + IV	42	18.50(15.95–22.92)	
**PNI**			
Absent	84	16.9(15.50–19.65)	**0.041**
Present	14	19.8(17.42–23.80)	
**HPR**			
**Necrosis**			
Absent	69	0.063(0.0401–0.086)	**0.015**
Present	29	0.041(0.029–0.068)	
**Tumor configuration**			
Ulcerated	27	0.054(0.04–0.07)	0.671
Ulceroinflitrative	71	0.051(0.03–0.08)	
**Tumor depth**			
T1 + T2	25	0.075(0.057–0.112)	**0.001**
T3 + T4	73	0.050(0.035–0.076)	
**Stage**			
I + II	56	0.0661(0.039–0.086)	0.126
III + IV	42	0.0478(0.038–0.073)	
I	17	0.080(0.046–0.120)	**0.008**
III	38	0.049(0.0381–0.073)	
**LNM**			
Absent	78	0.05(0.03–0.08)	0.341
Present	20	0.04(0.03–0.06)	
**LVI**			
Absent	32	0.075(0.049–0.093)	**0.013**
Present	66	0.049(0.037–0.074)	
**Distant Metastasis**			
Absent	95	0.05(0.03–0.08)	0.236
Present	3	0.03(0.03–0.04)	
**Tumor Size**			
<3 cm	42	0.069(0.041–0.091)	**0.015**
3 and above	43	0.051(0.039–0.078)	
**CEA (ng/mL)**			**0.033**
0–3	37	0.049(0.037–0.071)	
>3	61	0.063(0.040–0.089)	
**PLR**			
**Necrosis**			
Absent	69	150.90(103.40–299.52)	
Present	29	258.33(160.00–547.50)	**0.010**
**LNM**			
Absent	78	177(108.4–320.7)	0.172
Present	20	235(134.7469.3)	
**Tumor depth**			
T1 + T2	25	214(120.8–455.8)	0.634
T3 + T4	73	188.7(116.5–307.1)	
**Distant Metastasis**			
Absent	95	184(122.85–314.00)	
Present	3	705(245.12–889.32)	**0.0131**
**Stage**			
I + II	56	177(177–395.4)	0.851
III + IV	42	207(119–306.3)	
**NLR**			
**TALNR**			
Not seen	36	2.96(1.881–7.939)	**0.048**
Seen	62	6.15(2.247–16.700)	
**Tumor depth**			
T1 + T2	25	8.8(2.15–17.23)	0.276
T3 + T4	73	4.8(2.1–10.54)	
**Stage**			
I + II	56	5.54(2.18–15.46)	0.456
III + IV	42	5.04(1.95–11.07)	
**Tumor site**			
RC	24	4.5(2.11–14.72)	**0.006**
TC	**8**	**15.02(6.85–21.00)**	
LC	**24**	**3.52(1.86–9.09)**	
RS	13	5.33(2.05–24.91)	
R	29	4.87(1.93,11.05)	
**LNM**			
Absent	78	4.98(2.06–10.96)	0.177
Present	20	7.54(2.37–19.81)	
**Tumor configuration**			
Ulcerated	27	5.83(2.11–16.80)	0.694
Ulceroinflitrative	71	5.00(2.00–11.25)	

PLR: platelet to lymphocyte ratio; RDW: red cell distribution width; NLR: neutrophil-to-lymphocyte ratio; CEA: carcinoembryonic antigen; CA 19–9: carbohydrate antigen 19–9.

**Table 7:** Defining the cut-off values of PLR, NLR, RDW, and HPR, used alone or in combination, to distinguish colon cancer from healthy controls.

Parameters	J	Cut-Off	Sensitivity (%)	Specificity (%)	+LR	−LR	AUC (95% CI)
**PLR**	0.61	>105.574	79.6	81.6	4.33	0.25	0.842 (0.796–0.900)
**NLR**	0.50	>3.34	59.18	90.82	6.44	0.45	0.782 (0.720–0.840)
**RDW**	0.66	>15.7	73.5	92.9	10.29	0.29	0.876 (0.561–0.755)
**HPR**	0.45	≤0.0781	72.45	73.47	2.73	0.38	0.796 (0.747–0.861)
**NLR + RDW**	0.67	>0.4286	80.61	86.73	6.08	0.22	0.879 (0.825–0.921)
**PLR + NLR**	0.53	>0.4761	67.3	85.7	4.71	0.38	0.787 (0.401–0.612)
**PLR + RDW**	0.67	>0.4631	77.6	89.8	7.60	0.25	0.891 (0.561–0.744)
**PLR + NLR + RDW**	0.73	>0.4223	84.69	88.78	7.55	0.17	0.910 (0.860–0.943)

**Table 8:** Univariate survival analysis of clinicopathological parameters.

Parameters	*N*	3-Year OS	*Chi2*	*p*-Value	3-Year DFS	*Chi2*	*p*-Value
**Tumor Site**							
Colon	56	91.10%			73.20%		
Rectosigmoid	13	100%	3.9	0.14	61.50%	0.51	0.77
Rectum	29	82.80%			79.30%		
**Tumor Grade**							
WD	15	93.30%			66.70%	3	0.223
MD	69	88.40%	1.88	0.39	79.70%		
PD	14	92.90%			50%		
**LVI**							
Absent	32	96.40%	0.93	0.33	84.40%	1.6	0.307
Present	66	86.40%			68.20%		
**Node Status**							
Present	41	80.70%	6.4	**0.011**	53.70%	14.2	**0.01**
Absent	57	96.50%			87.70%		
**PNI**							
Present	14	71.70%	5.7	**0.017**	42.90%	6.5	**0.011**
Absent	84	92.50%			77.40%		
**LNM**							
Absent	78	94.90%	7.8	**0.005**	84.60%	19.3	**0.001**
Present	20	70%			30%		
**Tumor Depth**							
T1 + T2	25	90.90%			86.30%		
T3 + T4	73	66.70%	0.702	0.402	51.60%	14.47	0.074
**Stage**							
I + II	56	98.90%	8.72	**0.003**	89.30%	13.2	**0.001**
III + IV	42	75.70%			52.40%		
***SuFu* Expression**							
Low	62	83.80%	5.22	**0.023**	62.70%	8.15	**0.003**
Same as normal	36	100%			91.70%		
**CA 19.9**							
≤35	66	89.40%	0.94	0.331	78.80%	8.09	**0.004**
>35	32	90.60%			62.50%		

**Table 9:** Overall survival and disease-free survival analyses using the Cox proportional hazard model.

OS				DFS		
Parameters	H.R	95% CI	*p*-Value	H.R	95% CI	*p*-Value
Tumor site	0.44	0.05–3.6	0.451	1.4	0.38–5.5	0.573
PLR	3.1	0.63–15.65	0.162	0.6	0.25–1.72	0.404
NLR	0.2	0.05–1.24	0.090	1.5	0.61–3.94	0.404
RDW	0.310	0.510–1.763	0.187	1.4	0.33–6.53	0.601
CA 19.9	**2.06**	0.438–9.75	0.397	**3.07**	1.32–7.11	**0.009**
CEA	3.5	0.71–17.41	0.120	1.3	0.574–3.060	0.511
Tumor grade	2.1	0.03–6.3	0.052	1.3	0.34–5.4	0.658
LVI	3.8	0.32–45.5	0.281	0.93	0.28–3.06	0.911
Node status	0.8	0.21–3.7	0.870	1.02	0.21–4.9	0.98
PNI	1.06	0.08–13.59	0.936	1.8	0.64–5.1	0.260
Tumor depth	0.031	1.23–2.63	0.082	1.21	1.16–3.65	0.095
Stage	4.23	0.67–18.3	0.089	3.09	0.62–15.2	0.166
*SuFu* expression	2.9	0.83–10.45	0.093	0.34	0.10–1.13	0.080

**Table 10:** Correlation of blood biomarkers with *SuFu* expression.

Lab Parameters	*SuFu* Expression				
	Low	Same as Normal	Odds Ratio	*Chi2*	*p*-Value
**RDW**					
<15	10	4		0.008	0.928
≥15	59	25	1.01 (0.30–3.69)		
**PLR**					
<150	24	12	0.75 (0.31–1.75)	0.38	0.536
≥150	45	17			
**HPR**					
<0.07	55	19		2.23	0.136
≥0.07	14	10	2.06 (0.78–5.42)		
**NLR**					
<5	33	16	0.74 (0.31–1.78)	0.44	0.501
≥5	36	13			
**CEA (ng/mL)**					
0–3	30	7	2.4 (0.91–6.41)	3.25	0.071
>3	39	22			
**CA 19.9 (IU/mL)**					
≤35	44	22	0.56 (0.21–1.49)	1.35	0.244
>35	25	7			

## Data Availability

Data are available upon reasonable request.
